# Calidad de las imágenes, la lectura y el servicio de mamografía en cuatro centros de imagenología de Manizales, Colombia

**DOI:** 10.7705/biomedica.5135

**Published:** 2020-09-03

**Authors:** Karol Julieth García, Julián David Ocampo, María del Pilar Pardo, Tatiana Aguilar, Carlos Alberto Ruiz, Andrés Castaño

**Affiliations:** 1 Centro de Innovación, Investigación, Desarrollo y Transferencia de Tecnología, Facultad de Ingeniería, Universidad de Caldas, Manizales, Colombia; 2 Departamento de Informática y Computación, Universidad Nacional de Colombia, Manizales, Colombia

**Keywords:** mamografía, gestión de la calidad, diagnóstico por imagen, garantía de la calidad de atención de salud, servicio de mantenimiento e ingeniería en hospital, ingeniería biomédica, Mammography, quality management, diagnostic imaging, quality assurance, health care, maintenance and engineering, hospital, biomedical engineering

## Abstract

**Introducción:**

La calidad de la mamografía está directamente relacionada con la capacidad para detectar anormalidades y, por ello, es necesario el control de calidad en los centros de imágenes diagnósticas.

**Objetivo:**

Evaluar la calidad de la imagen, la lectura y el servicio de mamografía de algunos centros de imágenes diagnósticas en Manizales, Colombia.

**Materiales y métodos:**

Cuatro centros participaron de forma voluntaria y bajo acuerdos de confidencialidad en el estudio. De las 520 mujeres atendidas en ellos, a 318 se les hicieron mamografías. A partir de una inspección visual del servicio, se evaluaron la infraestructura, la tecnología y el personal de la unidad. Un radiólogo experto en lectura e interpretación clínica de imágenes mamarias evaluó la calidad de la imagen y la de su lectura. El análisis estadístico se hizo utilizando un anova y determinando el índice kappa y el porcentaje de desacuerdo.

**Resultados:**

Se encontró falta de calidad de las imágenes obtenidas, principalmente, por presencia de artificios en el 75% de ellas, e identificación y rotulación deficientes; además, en la toma de la proyección oblicua medio-lateral, se encontró falta de visualización del ángulo inframamario. El grado de concordancia en el reporte BI-RADS fue bajo en los cuatro centros, con diferencias importantes en el informe y la descripción de los hallazgos.

**Conclusión:**

Los centros de imágenes diagnósticas evaluados están habilitados para el funcionamiento, pero se encontraron deficiencias importantes en la calidad de las imágenes y en su lectura, lo que pone de manifiesto la necesidad de establecer estándares de calidad y mejorar los aspectos que se puedan mejorar.

## Abstract (en)

**Introduction:**

Mammography quality is directly related to the ability to detect an abnormality and, therefore, quality control is necessary for diagnostic imaging centers.

**Objective:**

To evaluate image quality, reading, and mammography service in some diagnostic imaging centers in Manizales, Colombia.

**Materials and methods:**

Four diagnostic imaging centers participated voluntarily in the study under confidentiality agreements. Out of 520 women attending the centers, 318 had a mammography. The infrastructure, technology, and human resources of each unit were evaluated based on visual inspections. A radiologist expert in reading and clinical interpretation of mammary images evaluated the quality of the image and the reading. We made the statistical analysis using anova, the kappa index, and the percentage of disagreement.

**Results:**

We found images of diminished quality mainly due to the presence of artifacts in 75% of those evaluated, as well as non-compliance with identification criteria and image labeling. There were difficulties in taking the lateral median oblique projection given the absence of the inframammary. The level of agreement in the BI-RADS reporting was low in the four centers with important differences in the report and description of findings.

**Conclusion:**

The city's diagnostic centers under evaluation are authorized for their operation. However, there are important deficiencies in image quality and reading, which highlights the need to seek quality standards starting from those aspects that can be improved upon.

El cáncer de mama constituye la principal causa de muerte por cáncer en mujeres a nivel mundial. En el 2018, representó el 25% de todos los cánceres, con una tasa de mortalidad del 13,0% y una tasa de incidencia del 46,3% por cada 100.000 mujeres, lo cual lo convierte en el tipo de cáncer más frecuente en las mujeres en los países desarrollados y en los menos desarrollados (1). En ese año, y a nivel mundial, Latinoamérica y el Caribe registraron el 27% del número de casos nuevos y el 16% de las muertes causadas por cáncer de mama, por lo cual este se consideró el principal tipo de cáncer en aumento y un problema creciente de salud en estas regiones (1-3). En el 2012, en Colombia, murieron 2.865 mujeres por cáncer de mama, cifra que en el 2016 aumentó a 3.013 muertes (4). Según el Departamento Administrativo Nacional de Estadística (DANE), en Caldas se reportaron 104 muertes por cáncer de mama en el 2016 (4).

En el reporte “Garantía de calidad de los servicios de mamografía: normas básicas para América Latina y el Caribe” (3), la Organización Panamericana de la Salud (OPS) y la Organización Mundial de la Salud (OMS) establecieron que la mamografía es el estudio de imagenología más eficiente para la tamización del cáncer de mama, dada su capacidad para detectar lesiones en las fases preclínicas. Sin embargo, la mamografía es una de las exploraciones radiológicas más exigentes debido, entre otros aspectos, a la alta resolución, el contraste y el rango dinámico que requiere (5). Esto ha suscitado polémicas debido al riesgo que la irradiación implica para las mujeres asintomáticas, así como por los falsos positivos y falsos negativos que pueden producirse (6).

En Colombia, la mamografía se considera la herramienta de tamización más importante para el cáncer de mama (7) y diversos estudios clínicos, como el *Health Insurance Plan* (HIP), han demostrado su impacto, con una disminución de la mortalidad hasta del 30% (8). En la actualidad, diferentes sociedades científicas recomiendan practicarla cada dos años con fines de tamización en mujeres de 50 a 69 años de edad.

En investigaciones previas se ha demostrado que la calidad de la imagen y de la lectura de la mamografía están directamente relacionadas con la capacidad de detección de anormalidades (9), pues las imágenes de baja calidad, además de dificultar la interpretación, incrementan el número de exámenes adicionales (ultrasonido y biopsias), lo que genera ansiedad innecesaria en las pacientes y disminuye el valor de la mamografía como prueba de tamización (10). Otros autores han concluido que la mamografía sin un control de calidad adecuado disminuye la sensibilidad en el 66%, en tanto aquellas con un control adecuado alcanzan una sensibilidad del 85 al 90% y una especificidad de 81,8% (11-13).

Asimismo, se ha evidenciado la necesidad del control de calidad de la imagen mamográfica y su interpretación en los centros de imágenes diagnósticas. Según Cataliotti, *et al.* (14), uno de los criterios para la evaluación de los programas de tamización son las condiciones de trabajo de los radiólogos, en especial, el número de profesionales por centro, las mamografías que deben realizar por año, el tiempo de dedicación y la capacitación en esquemas de control de la calidad radiológica. Por su parte, Koch, *et al.* (15), demostraron en su estudio que el personal de salud encargado de la lectura de las mamografías en el servicio público de salud en Río de Janeiro no tenían el conocimiento suficiente para brindar un servicio de calidad, lo que limitaba un diagnóstico oportuno y confiable. En otros estudio, se evaluaron la imagen mamográfica y la calidad del reporte en 165 unidades de servicio de salud de Turquía, y se evidenciaron deficiencias significativas en términos de calidad, por lo que se sugirió invertir más tiempo y esfuerzo en capacitar a los radiólogos y técnicos en radiología, con el fin de garantizar imágenes e interpretaciones acordes con los estándares y criterios de calidad establecidos internacionalmente (16).

Es claro, entonces, que los servicios de mamografía deben contar con un programa que garantice la calidad de la imagen, de la lectura y del servicio, sin poner en riesgo la seguridad de las pacientes, de manera que se obtenga la información necesaria para un diagnóstico preciso que permita el tratamiento oportuno. Claramente, ello implica invertir para tener personal calificado y capacitado, así como la tecnología apropiada para la prestación del servicio. De ahí la necesidad de evaluar la calidad de la imagen, la lectura y el servicio de algunos centros de imágenes diagnósticas de Manizales (Caldas), para determinar y analizar el estado actual de los centros, y proponer planes y programas de seguimiento, evaluación y control del servicio.

## Materiales y métodos

Se hizo un estudio analítico, experimental y prospectivo en cuatro centros de imágenes diagnósticas (mamografías digitales y digitalizadas), que atienden a mujeres afiliadas al régimen subsidiado y provenientes de diversos municipios del departamento de Caldas. De 520 mujeres seleccionadas en un programa previo de tamización de cáncer de mama en diferentes municipios del departamento, se les hicieron mamografías a 318 de ellas escogidas según los criterios de inclusión en el estudio. Los centros diagnósticos evaluados aceptaron su participación en la investigación de forma voluntaria y bajo acuerdos de confidencialidad.

### Criterios de inclusión

Se seleccionaron mujeres mayores de 50 años con examen clínico negativo de mama y que no se hubieran practicado mamografías con fines de tamización en los últimos 12 meses. Además, se incluyeron mujeres mayores de 40 años con alguna alteración en el examen clínico de mama, indiferentemente de si se hubieran practicado o no una mamografía diagnóstica en los últimos 12 meses.

### Criterios de exclusión

Se excluyeron las mujeres con examen clínico negativo de mama sometidas a mamografía en los últimos 12 meses y las menores de 50 años también con examen clínico negativo.

En función de los criterios de evaluación de los programas de tamización (7), entre ellos, la efectividad de la mamografía como prueba de tamización, los daños y riesgos de la tamización con mamografía, la edad de inicio de la tamización con mamografía, las recomendaciones de las guías de práctica clínica y las consideraciones éticas relativas a las pruebas de tamización, se evaluó la calidad del servicio de mamografía en cada uno de los centros, estableciendo categorías según la calidad de la imagen, de la lectura y del servicio.

Para el desarrollo del estudio, fueron necesarias la parametrización y la sistematización del protocolo de evaluación de calidad de la imagen y el reporte del diagnóstico, utilizando el programa de seguimiento diseñado para la investigación y con base en la quinta edición del sistema de clasificación internacional *Breast Image Reporting and Data System* (BI-RADS) (17). Cada centro de diagnóstico ingresó en dicho programa las imágenes tomadas y generó un reporte de diagnóstico por paciente. Fue necesario transcribir los reportes según la parametrización establecida, ya que una variable de análisis fue la inclusión de todos los criterios de diagnóstico aceptados internacionalmente para los reportes mamográficos.

Un radiólogo experto en lectura e interpretación clínica de imágenes mamarias evaluó cada una de las imágenes ingresadas en el programa de seguimiento sin conocer el reporte emitido por el centro y generó uno nuevo por paciente (reporte de referencia).

Para la evaluación de la calidad de la imagen, se tomaron los parámetros considerados de mayor impacto por el radiólogo experto de acuerdo con las guías de control de calidad de la mamografía digital y su práctica clínica (18,19): calidad de la visualización, identificación de la paciente y proyecciones, calidad de la imagen en la proyección oblicua medio-lateral derecha e izquierda según los criterios de correcta identificación y rotulación de la imagen, exposición apropiada, comprensión adecuada de la necesidad de mantener el pecho firme y sin movimiento, imagen nítida, pezón de perfil, músculo pectoral a nivel del pezón, visualización clara del ángulo inframamario, imagen de mama completa e imágenes simétricas. Asimismo, se incluyó la calidad de la imagen en la proyección cráneo-caudal derecha e izquierda bajo los siguientes criterios: correcta identificación y rotulación de la imagen, exposición apropiada, comprensión adecuada, imagen nítida y sin presencia de artefactos ni pliegues en la piel, visualización del pezón y del borde medial, imagen de la mama completa, ausencia de artefactos e imágenes simétricas.

En cuanto a la calidad de la lectura de la imagen, se tomó como referencia el resultado del radiólogo experto y, posteriormente, se comparó uno a uno cada reporte con base en los criterios establecidos para el BI-RADS, con el fin de detectar las diferencias en los reportes mamográficos.

La calidad del servicio se evaluó según las características tecnológicas, clínicas y humanas utilizando un protocolo adaptado a partir de la revisión meta-narrativa (20) de García-Luna, *et al.*, y las guías de control de calidad (3,21), las cuales incluyen: identificación del equipo, número de mamografías por día, programa de tecnovigilancia y mantenimiento, periodicidad de los estudios radiofísicos, número de profesionales especializados en el área, capacitación del personal, aplicación de las normas de radioprotección, dotación del servicio, tiempos de comunicación de los resultados y de asignación de las citas, e infraestructura.

No se incluyó la medición de la dosimetría como parámetro de la calidad del servicio, porque no se contaba con el dispositivo de medición necesario, y se asumió que este había sido evaluado directamente en el momento de los estudios radiofísicos. La dosimetría es un parámetro fundamental que debe evaluarse en cualquier estudio de calidad, pues permite establecer qué tan bien calibrado está el equipo en el momento de estimar la dosis glandular promedio para diferentes mamas (21,22).

Para evaluar la calidad de la imagen y del servicio, se analizó la información obtenida mediante estadística descriptiva, un anova con un factor de confianza del 95%, el índice kappa aplicado a las variables del reporte de lectura mamográfica y el índice de desacuerdo en la lectura entre los centros diagnósticos y el radiólogo experto con la herramienta estadística SPSS™. El índice kappa se interpretó con base en la escala de valoración propuesta por Landis, *et al.*, y Abraira (23,24), como se muestra en el cuadro 1.

**Cuadro 1:** Valoración del índice kappa. Valores de referencia tomados para determinar la concordancia entre los observadores

**Índice kappa**	**Fuerza de la concordancia**
Menor de 0,00	Sin acuerdo
0,00 a 0,20	Insignificante
0,21 a 0,40	Media
0,41 a 0,60	Moderada
0,61 a 0,80	Sustancial
0,81 a 1,00	Casi perfecta

Con base en el anova de un factor, se establecieron las siguientes hipótesis: H_0_, no hay diferencia significativa entre los grupos, y H_a_, hay diferencias significativas entre ellos.

Así, si el valor de p era mayor de 0,05, se aceptaba la hipótesis nula (H_0_); por el contrario, si el valor de p era menor de 0,05, se rechazaba la hipótesis nula (H_0_) y se aceptaba la hipótesis alternativa (H_a_).

La relación entre las pruebas estadísticas utilizadas en el estudio se presenta en el cuadro 2.

**Cuadro 2:** Descripción de la relación de los resultados de cada una de las pruebas estadísticas utilizadas en el estudio: valor kappa, índice del anova de un factor e índice de desacuerdo

**Índice kappa**	**Índice del anova de un factor**	**Índice de desacuerdo**
Moderado Sustancial Casi perfecto	p mayor de 0,05: se acepta la hipótesis nula.	Porcentaje menor de 10%
Sin acuerdo Insignificante Medio	p menor de 0,05: se rechaza la hipótesis nula.	Porcentaje mayor de 10%

## Resultados

De las 520 pacientes seleccionadas para el estudio, la mamografía se hizo finalmente en 318 mujeres (mamografías digitales y digitalizadas) distribuidas de la siguiente forma: centro 1 (C1), 88 mamografías; centro 2 (C2), 74 mamografías; centro 3 (C3), 91 mamografías, y el centro 4 (C4), 65 mamografías.

### Calidad de la imagen

Teniendo en cuenta los criterios seleccionados para la evaluación y el puntaje otorgado por el radiólogo experto, se encontró que, en la categoría de calidad de la visualización, el 75% de las imágenes presentaba artificios, por lo cual no cumplían con los estándares establecidos en las guías internacionales y evaluados por el radiólogo. Por otro lado, en la categoría de identificación y rotulación de la imagen, en ninguno de los centros se cumplía con la identificación del técnico a cargo del procedimiento ni con la de la organización.

En la categoría de calidad de las imágenes, en la proyección oblicua medio-lateral derecha, todos los centros presentaban una correcta identificación y rotulación de la imagen, la imagen completa de la mama, imágenes simétricas y una compresión adecuada, lo que se comprueba con una imagen sin movimiento y nítida y una exposición de radiación adecuada. Sin embargo, el 94% de las imágenes evaluadas en el estudio presentaba artificios que oscurecían las imágenes, y el 82,01% de estas no mostraba claramente el ángulo inframamario, en tanto que, en uno de los centros, el 69% de las imágenes presentaba pliegues oscuros en la piel en la proyección oblicua medio-lateral derecha y, el 66%, en la izquierda.

En la proyección oblicua medio-lateral izquierda, en todos los centros hubo una correcta identificación y rotulación de la imagen, una exposición y compresión adecuadas de la mama, imagen nítida y simetría en las imágenes, visualización del pezón, así como la imagen de la mama completa. Sin embargo, el 92% de las proyecciones oblicuas evaluadas de la mama izquierda presentó artefactos que las oscurecían y el 86% no cumplía con los criterios mínimos de visualización del ángulo inframamario.

En la categoría de calidad de la imagen en la proyección cráneo-caudal derecha e izquierda, todos los centros participantes cumplieron con los rangos mínimos de calidad establecidos en las guías para cada una de sus variables: correcta identificación y rotulación de la imagen, exposición apropiada y compresión adecuadas, imagen nítida, imagen de mama completa, y ausencia de artefactos, entre otros.

### Calidad de lectura

En el cuadro 3 se presentan los resultados obtenidos tras el análisis estadístico mediante el índice kappa de concordancia, el anova de un factor y el porcentaje de desacuerdo con base en los datos obtenidos en el trabajo de campo.

**Cuadro 3:** Resultados de cada centro de imágenes según los principales criterios de calidad de la lectura de la imagen mamográfica establecidos en el estudio

**Composición mamaria**			
**Centro de diagnóstico**	**Índice kappa**	**Índice del anova de un factor**	**Índice de desacuerdo**
C1	0,724	0,6019	4,5
C2	0,318	0,0960	4,1
C3	0,403	0,0740	25,0
C4	0,334	0,0013	21,9
**Existe masa**			
**Centro de diagnóstico**	**Índice kappa**	**Índice del anova de un factor**	**Índice de desacuerdo**
C1	0,327	0,000260633	20,5
C2	0,602	0,999999819	10,8
C3	0,175	0,238135452	31,9
C4	0,516	0,295821213	15,6
**Forma de la masa**			
**Centro de diagnóstico**	**Índice kappa**	**Índice del anova de un factor**	**Índice de desacuerdo**
C1	-0,135	0,155614956	85,71
C2	-0,111	1	62,5
C3	0,146	0,002121124	71,4
C4	-0,286	0,188998593	66,6
**Márgen de la masa**			
**Centro de diagnóstico**	**Índice kappa**	**Índice del anova de un factor**	**Índice de desacuerdo**
C1	0,063	0,007974464	85,71
C2	0,429	0,316979253	25,0
C3	-0,2	0,599867373	85,7
C4	-0,2	0,130932801	66,6
**Existe asimetría**			
**Centro de diagnóstico**	**Índice kappa**	**Índice del anova de un factor**	**Índice de desacuerdo**
C1	0,097	0,013243586	18,2
C2	-0,21	0,313958542	5,4
C3	0,179	0,299705705	19,8
C4	0,122	0,00036924	30,8
**Distorsión del parénquima sin masa visible**			
**Centro de diagnóstico**	**Índice kappa**	**Índice del aqnova de un factor**	**Índice de desacuerdo**
C1	0,044	4,53146E-13	17,0
C2	0	8,0187E-14	48,7
C3	0,017	0,314597456	4,4
C4	NA	NA	0,0
**Presencia de calcificaciones**			
**Centro de diagnóstico**	**Índice kappa**	**Índice del anova de un factor**	**Índice de desacuerdo**
C1	0,131	3,19121E-11	46,6
C2	0,456	0,080078509	24,3
C3	0,131	7,7032E-06	22,0
C4	0,618	0,853522694	18,5
**Tipo de morfología de la calcificación**			
**Centro de diagnóstico**	**Índice kappa**	**Índice del anova de un factor**	**Índice de desacuerdo**
C1	0,476	0,309552041	6,1
C2	NA	NA	0,0
C3	0,549	0,700602226	4,3
C4	0	0,155863942	5,3
**Distribución de las calcificaciones**			
**Centro de diagnóstico**	**Índice kappa**	**Índice del anova de un factor**	**Índice de desacuerdo**
C1	0,05	1,04253E-34	77,3
C2	0,04	6,66932E-22	68,1
C3	0,009	2,63482E-33	76,9
C4	0,227	6,71366E-07	46,2
**Categoría BI-RADS**			
**Centro de diagnóstico**	**Índice kappa**	**Índice del anova de un factor**	**Índice de desacuerdo**
C1	0,093	3,95035E-11	38,4
C2	0,275	0,008858293	16,2
C3	0,269	0,000312655	36,3
C4	0,073	0,001912098	38,5

NA: no aplica

### Análisis por subgrupos

*Centro 1.* El análisis de los resultados en la lectura de la mamografía comparada con la del experto, no reveló diferencias estadísticamente significativas en las variables de composición mamaria (grado de acuerdo sustancial) y tipo de morfología de la calcificación (grado de acuerdo moderado) y un porcentaje de desacuerdo menor del 10% en ambas variables. En el resto de las variables, con excepción de la forma de la masa (no evaluable), hubo diferencias estadísticamente significativas con un grado de acuerdo insignificante.

*Centro 2.* El análisis de los resultados de la lectura de la mamografía comparada con la del experto, no evidenció diferencias estadísticamente significativas en las variables de existencia de masa, margen de esta y presencia de calcificaciones (grado de acuerdo moderado). Las variables de distribución del parénquima y las calcificaciones y el reporte BI-RADS final presentaron diferencias estadísticamente significativas con un grado de acuerdo insignificante. El resto de las variables no fueron evaluables.

*Centro 3*. El análisis de los resultados de la lectura de la mamografía comparada con la del experto, no evidenció diferencias estadísticamente significativas en la variable de tipo de morfología de la calcificación, con un grado de acuerdo sustancial y un porcentaje de desacuerdo de 4,3%. Las variables de forma de la masa, presencia y distribución de las calcificaciones y el reporte BI-RADS final presentaron diferencias estadísticamente significativas, con un grado de acuerdo insignificante. El resto de las variables no fueron evaluables.

*Centro 4*. El análisis de los resultados de la lectura de la mamografía comparada con la del experto, no registró diferencias estadísticamente significativas en la variable de existencia de masa (grado de acuerdo moderado) y presencia de calcificaciones (grado de acuerdo sustancial). Las variables de composición mamaria, existencia de asimetría, distribución de las calcificaciones y reporte BI-RADS final presentaron diferencias estadísticamente significativas con un grado de acuerdo insignificante. El resto de las variables no fueron evaluables.

### Calidad de servicio

Según la información suministrada por los centros y tras la evaluación tecnológica, técnica y humana, se encontró que el 50% de los equipos eran digitales, en tanto que el otro 50% contaba con un dispositivo para su digitalización. El 25% de los equipos evaluados tenían más de 10 años de servicio. El 75% de los centros realizaba menos de 30 mamografías al día.

En cuanto a la categoría de tecnovigilancia y mantenimiento, los cuatro centros contaban con licencia de funcionamiento. A nivel estructural, los servicios incluían una sala exclusiva para el equipo, un área de control, un área para cambio de ropa de las pacientes y un área para la lectura de las placas, y disponían de unidad sanitaria. A nivel administrativo, todos los equipos contaban con la hoja de vida ajustada al formato del Invima y a las pautas establecidas por el Ministerio de Salud y Protección Social. Asimismo, en todos los casos el mantenimiento preventivo estaba a cargo de empresas externas y se llevaba a cabo cada tres a seis meses.

En cuanto a los estudios radiofísicos, se encontró que en todos los centros se hacían cada 12 a 48 meses. Asimismo, se cumplían las condiciones de radioprotección establecidas a nivel nacional para la habilitación: paredes y puertas de la sala de mamografía blindadas con plomo, dotación apropiada para servicios de mediana complejidad (delantal plomado y protector de tiroides), personal capacitado para la práctica en imagenología, manual de radioprotección y un protocolo de garantía de la calidad de la imagen.

En los cuatro centros participantes, el personal involucrado en el servicio de mamografía (tecnólogo, radiólogo, jefe de enfermería y auxiliares) recibía capacitaciones semestrales a cargo de empresas externas. Los centros contaban, por lo menos, con dos tecnólogos en radiología capacitados en la toma de imágenes mamarias. Además, tres de los cuatro centros evaluados contaban con dos especialistas en radiología para la lectura de las mamografías y, uno, con cuatro especialistas. Todos reportaron haber realizado capacitaciones de mínimo 200 horas en la lectura de las mamografías y, al menos, 200 lecturas de imágenes mamográficas.

En cuanto a la asignación de citas y la comunicación de los resultados, todos los centros diagnósticos disponían de tres días hábiles para la asignación de las citas, y tres hacían seguimiento a las pacientes y reasignaban la cita en caso de presentarse problemas con la autorización del examen por parte de las empresas prestadoras de servicios de salud (EPS). En todos los centros, el tiempo máximo de duración del procedimiento era de 15 minutos, y disponían de tres días hábiles para la entrega del resultado.

Teniendo en cuenta la necesidad de estandarizar la terminología, organizar los informes y estructurar la evaluación para que los radiólogos comuniquen los hallazgos de manera clara y sucinta, en todos los centros se utilizaba el sistema BI-RADS de clasificación de la enfermedad, y, además, en caso de reportarse una categoría BI-RADS de 4 o 5, los centros disponían de un máximo de dos días hábiles para informarlo a la EPS y a la paciente.

### Discusión

El cáncer de mama es la primera causa de morbimortalidad por cáncer en las mujeres a nivel mundial, y el departamento de Caldas no es ajeno a esta situación. El impacto de la mamografía en la mortalidad específica por este cáncer se ha demostrado en varios estudios (6,7,25-27), ya que su adecuada realización, interpretación y cobertura afecta directamente este indicador.

En la guía para la práctica de imágenes de mama elaborada por el *Royal Australian and New Zealand College of Radiologists* (18) y la guía europea para el control de calidad (21), se establece que los informes de radiología de la mama deben ser sinópticos y estandarizados, y la información debe estar parametrizada para mejorar la comunicación entre los médicos tratantes y otros profesionales de la salud.

Esta es la primera publicación sobre la calidad de la lectura, la imagen y el servicio de las mamografías en Caldas (Colombia), y es un punto de partida para la elaboración de planes y programas de seguimiento, evaluación y control del servicio.

Según los criterios de evaluación, la calidad de las imágenes en los cuatro centros de diagnóstico participantes no es la idónea debido a la presencia de artificios; el déficit en la información que debe consignarse en las placas (fecha del estudio, nombre del centro diagnóstico, identificación del técnico radiólogo, número de identificación del mamógrafo cuando se cuenta con más de uno, datos de miliamperios por segundo (mA/s) y tensión (máxima: kV(p)); las dificultades para la toma adecuada de la proyección oblicua medio-lateral (exposición irregular por presencia de espacios, pliegues, visualización cóncava del borde muscular y forma triangular, o visualización paralela al borde de la placa del músculo pectoral, no visualización del pliegue inframamario y formación de pliegues en el lado de la rejilla de Bucky).

De todas maneras, al asociar estos datos con la calidad de los servicios, se evidencia que hay tecnólogos especialistas en la toma de imágenes mamográficas, pero que no se hace de forma constante la verificación inmediata por parte de los médicos radiólogos durante el procedimiento para garantizar la calidad de la imagen, lo que probablemente se refleja en la dificultad para mejorar las deficiencias en el posicionamiento y la exposición de la glándula mamaria, e influye en el aumento de falsos negativos o positivos y la creciente necesidad de citar de nuevo a las mujeres.

Estos resultados son similares a los de otros estudios que han resaltado la importancia de la retroalimentación del médico radiólogo intérprete al técnico radiólogo sobre la calidad de la imagen, la cual se ve afectada por los errores en la posición de la paciente, y las características de la densidad y el contraste, así como en la inadecuada exposición de la glándula mamaria (16,28,29).

Por otro lado, la presencia de artificios en la proyección oblicua medio-lateral en todos los centros, puede estar relacionada con el estado de los equipos, su longevidad y la digitalización en dos de ellos, pues, aunque están acreditados y licenciados ante el Ministerio de Salud y Protección Social y cuentan con la certificación de los estudios radiofísicos y de mantenimiento, hay razón para dudar sobre su idoneidad, ya que son procesos llevados a cabo por empresas externas que no están reguladas por el Ministerio. Es muy importante una evaluación regular de las imágenes mediante la acreditación Phantom diaria por parte del tecnólogo y el médico radiólogo, para garantizar la calidad de la imagen mamográfica (30), lo cual permitiría a los centros diagnósticos corregir las deficiencias en las imágenes provocadas por el equipo. En cuanto a la deficiencia en la identificación, este podría ser consecuencia de una falta de estandarización de los procesos internos de cada centro.

La calidad de la lectura se analizó con base en el sistema de informes y registro de datos de las imágenes de mama BI-RADS, el cual busca estandarizar el reporte mamográfico, y es una herramienta de investigación y seguimiento (31). Según el índice kappa, el nivel de concordancia en el reporte BI-RADS fue bajo y las concurrencias presentadas podrían deberse al azar. Las diferencias trascendentales evidenciadas en el reporte y la descripción de hallazgos, pueden alterar en algunos casos el reporte BI-RADS final como se muestra en el cuadro 3; esto concuerda con los resultados de diferentes estudios (10,15,16,28), en los que se ha reportado conocimiento insuficiente por parte de los radiólogos, déficit en la capacitación y la evaluación del conocimiento de los tecnólogos y radiólogos, y falta de concordancia en la clasificación de la enfermedad con respecto al enfoque recomendado.

Si bien la mamografía permite un diagnóstico oportuno del cáncer de mama, su interpretación debe tener buena calidad; sin embargo, esta depende de cada médico radiólogo, de su habilidad perceptiva y cognitiva, de un amplio conocimiento de los posibles errores que pueden darse al interpretar mamografías y de cómo prevenirlos, corregirlos o minimizarlos. Por ello, los radiólogos necesitan calificarse específicamente para la lectura de imágenes de la mama y cumplir con las horas de práctica, con el fin de verificar su nivel de conocimiento para la interpretación de imágenes (14). El grado de concordancia entre los radiólogos al interpretar la misma mamografía es bajo y hay una variación importante entre observadores y entre interpretaciones, lo que puede retrasar la detección del cáncer de mama o resultar en biopsias innecesarias, con consecuencias físicas, económicas y psicológicas indeseables (32).

Entre los factores causantes de la disminución de la calidad en la interpretación clínica de las imágenes, puede incluirse el reducido número de radiólogos con educación superior formal y certificada en imágenes mamarias que, además, no realizan todas las lecturas de cada centro, por lo que el número de mamografías leídas por los radiólogos podría variar significativamente e influir en la experiencia adquirida. Por otra parte, en la mayoría de los centros, la lectura de las imágenes es diferida, por lo cual las tomas complementarias no se solicitan durante la práctica del estudio inicial, lo cual aumenta el número de BI-RADS 0 y 3, y hace necesaria una nueva cita para las pacientes.

Aunque se hacen esfuerzos para aumentar la cobertura de la mamografía de tamización y de las campañas de educación sobre la detección temprana del cáncer de mama, es necesario garantizar el adecuado funcionamiento de las unidades de mamografía, ya que las deficiencias en el servicio, la calidad de la imagen y la lectura, influyen negativamente en la sensibilidad y la especificidad de la prueba. La inclusión de medidas de control de calidad del servicio, incluidas la evaluación y el cumplimiento de los criterios establecidos a nivel nacional e internacional, influiría positivamente en la atención y el tratamiento del cáncer de mama en los centros de atención (22).

Para lograr un impacto positivo en las estadísticas del cáncer de mama, es necesario contar con unidades que garanticen una mejor capacitación, actualización y entrenamiento de los tecnólogos especializados en la toma de mamografías, un adecuado mantenimiento de los equipos con pruebas de inspección de calidad, y personal especializado en radiología con la mayor experiencia posible en imágenes mamarias.

Este estudio puso de manifiesto las carencias de los centros de imágenes diagnósticas en Manizales y es un punto de partida hacia la meta de mejorar los estándares de calidad necesarios, optimizando los aspectos que se pueden mejorar, en aras de aumentar las tasas de detección de cáncer de mama y disminuir así la detección tardía de la enfermedad y la mortalidad de nuestras mujeres por esta causa. Los resultados de este estudio se comunicaron a cada uno de los centros participantes en un informe con las recomendaciones para mejorar la calidad.

### Limitaciones del estudio

No se pudieron correlacionar los datos estadísticos teniendo en cuenta su enfoque, que contribuiría a establecer el grado de concordancia, significancia y desacuerdo del estudio, por lo cual no se pudo hacer una interpretación en común.

Uno de los centros diagnósticos participantes incluyó los resultados de exámenes adicionales en su clasificación mamográfica, lo que impidió compararla con la del radiólogo experto que hizo la revisión, quien solo tenía acceso a las imágenes mamográficas. Este es el único centro en que se hacen estudios adicionales de forma inmediata y según la necesidad a partir de los resultados de la mamografía.
